# Supplementary material for: Trait-Based Vaccination of Individual Meerkats (Suricata suricatta) against Tuberculosis Provides Evidence to Support Targeted Disease Control
Source: Animals (Basel). 2022 Jan 13;12(2):192. doi: 10.3390/ani12020192 (PMC8772857; doi:10.3390/ani12020192)
Supplement: Supplementary file 1 [file animals-12-00192-s001.zip › animals-1537617-supplementary.pdf]

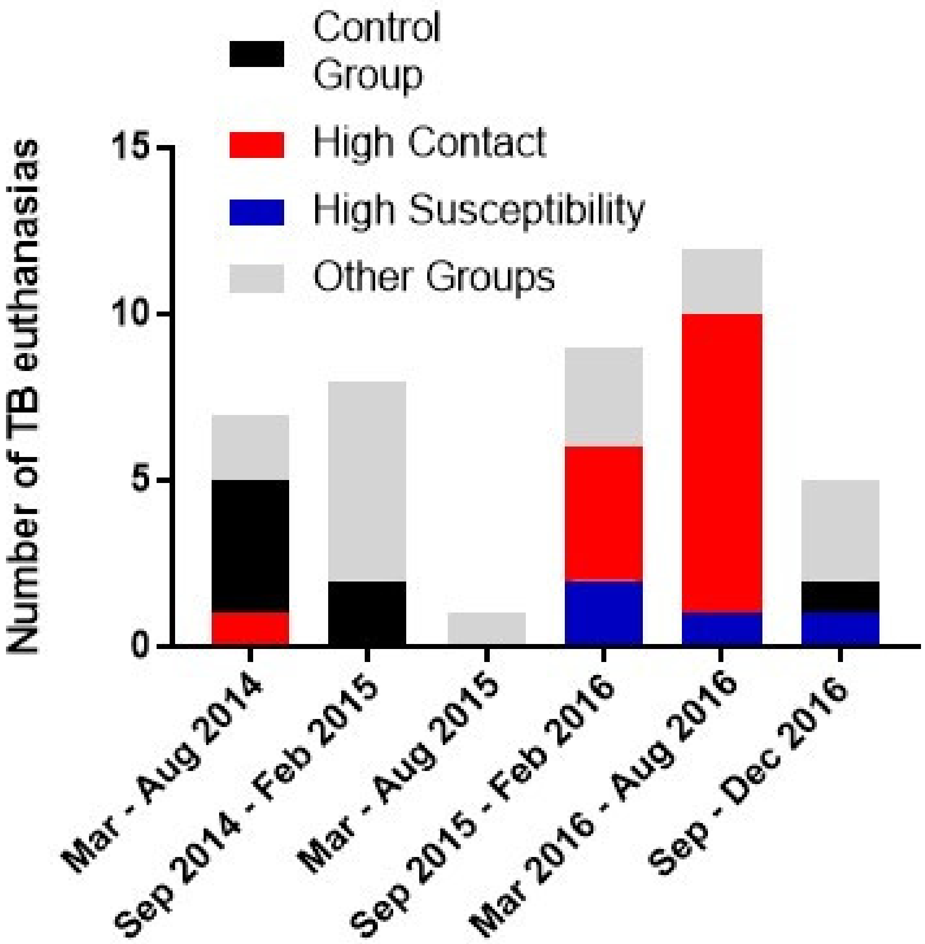

Figure S1. Clinical cases of tuberculosis at the KMP from 2014–2016.

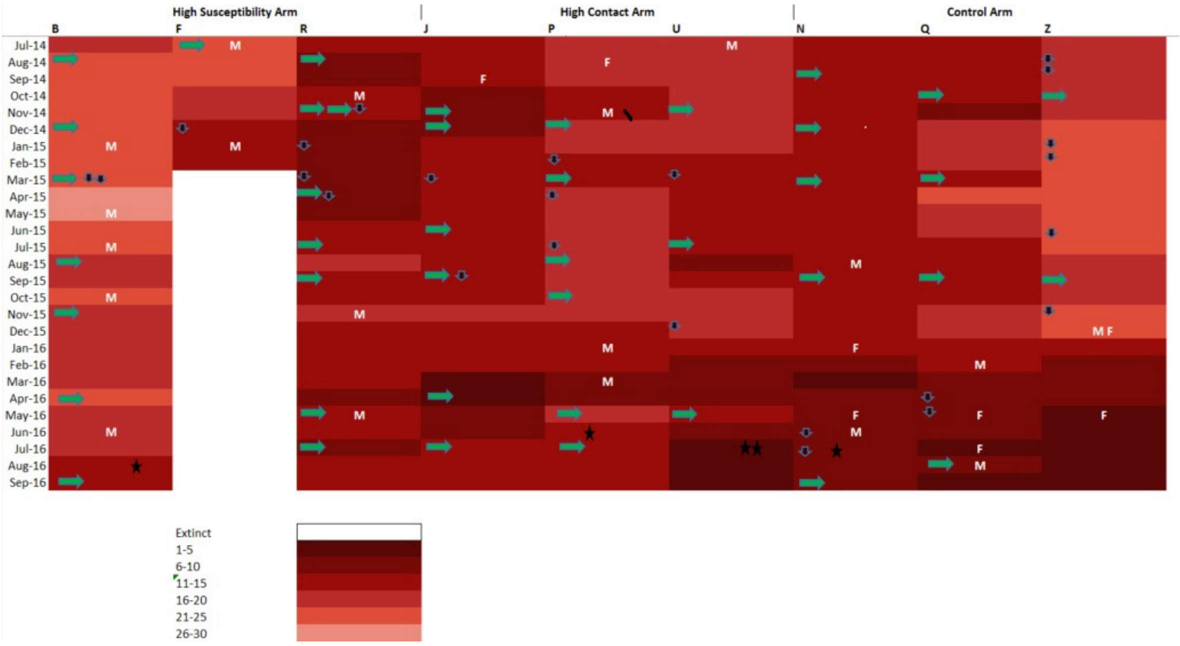

Figure S2. Group Changes throughout the study.

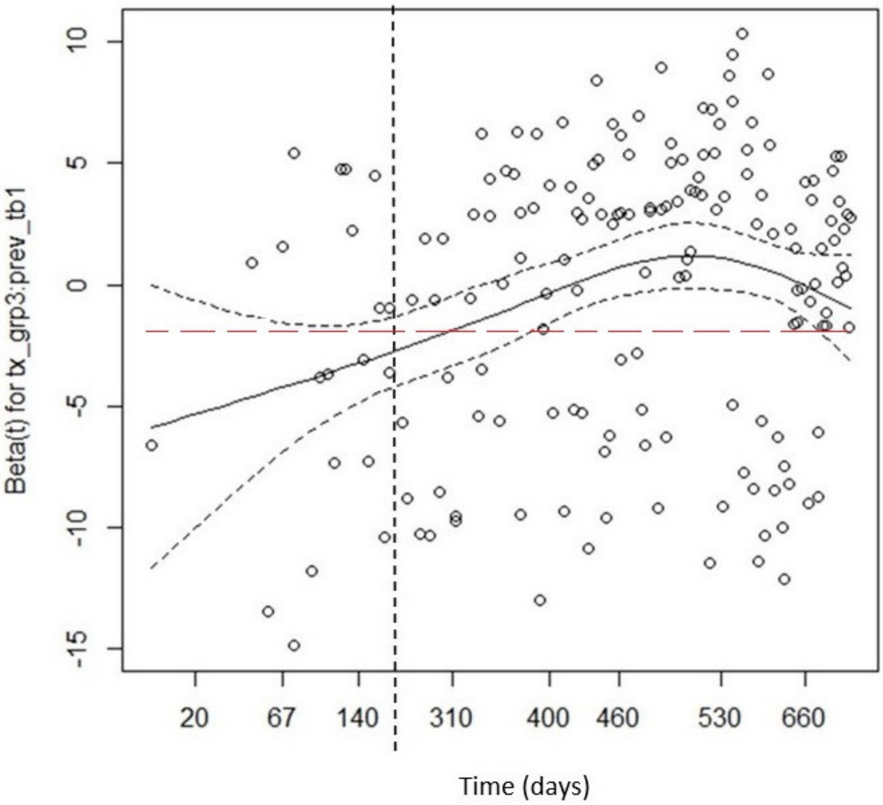

Figure S3. Scatterplot of scaled Schoenfeld residuals for the multivariable model of survival time to death.

Table S1. Treatment set composition at commencement of the study.

**Group**	**Treatment Set**	**Group Size Sep 2014**	**Group Composition**	**Previous Group History of TB**	**Initial TB Test positive prevalence (%)**
N	Control	9	1 DF, 1DM, 2 AF, 3 AM, 1SF, 1 SM	No	0.0
Q	Control	11	1 DF, 1 DM, 1 AF, 3 SF, 2 SM, 2 JF, 1 JM	No	33.3
Z	Control	20	1 DF, 1 DM, 4 AF, 9 AM, 2 SF, 2 SM, 1 JM	No	45.4
B	High Susceptibility	19	1 DF, 1DM, 9 AF, 4AM, 4SM	No	7.1
F	High Susceptibility	22	1 DF, 1DM, 3 JF, 4JM, 1 AF 8 AM, 2PF, 2PM	No	100.0
R	High Susceptibility	9	1 DF, 1 DM, 2 AF, 2 AM, 2 SF, 1 SM	Yes, last case in 2009	30.0
J	High Contact	9	1 DF, 1DM, 2 AF, 2 AM, 1SF, 2 SM	No	20.0
P	High Contact	16	1 DF, 1 DM, 1 AF, 2 AM, 6 SF, 5 SM	No	37.5
U	High Contact	20	1 DF, 1 DM, 4 AF, 6 AM, 3 SF, 2 SM, 3 JM	No	36.4

Table S2. Survival analysis for time to a positive test in 221 wild meerkats.

Variable		*HR **	*95% Confidence Interval*	*Wald Test p Value for Variable*	*HR **	*95% Confidence Interval*	*p Value*
Dominance	*No*			0.188			
	Yes	0.53	0.21–1.36				
Sex	*F*			0.043			
	M	1.87	1.02–3.44		**1.97**	**1.06–3.67**	**0.033**
Age	*0-6 months*			0.068			
	6-12 months	2.02	0.82–4.98		2.06	0.84–5.07	0.12
	12-24 months	2.15	1.01–4.56		**2.12**	**1.00–4.51**	**0.05**
	>24 months	0.74	0.22–2.43		0.73	0.22–2.42	0.61
Previous history of TB within social group	*No*			0.806			
	Yes	0.92	0.49–1.75				
Treatment Set	*Control*			0.92			
	High Susceptibility	1.14	0.51–2.55				
	High Contact	0.92	0.53–1.78				
Social group	Full list of groups not presented here			0.81	Variance of random effect <0.0001		

*HR, Hazard Ratio.
